# 2320. Impact of Lifting School Mask Mandates on Community COVID-19 Cases and Hospitalizations: A Nationwide, Retrospective Cohort Study with an Event Study Design

**DOI:** 10.1093/ofid/ofad500.1942

**Published:** 2023-11-27

**Authors:** Zeynep Ertem, Anesh Danesharasteh, Elissa Schechter-Perkins, Richard Nelson, Lloyd Fisher, Shira Doron, Westyn Branch-Elliman

**Affiliations:** SUNY Binghamton, Binghamton, New York; Binghamton, Binghamton, New York; Boston University School of Medicine, Boston, Massachusetts; University of Utah, Salt Lake City, Utah; Reliant Medical Group, UMass Medical Center, Worchester, Massachusetts; Tufts Medical Center, Boston, Massachusetts; VA Boston Healthcare System, West Roxbury, MA

## Abstract

**Background:**

During the school re-opening process, masking mandates were widely adopted in schools in the United States to reduce spread of SARS-CoV-2 in in-school settings and in the surrounding community. The aim of this nationwide, retrospective cohort with an event study design was to evaluate the impact of masking policy de-adoption on SARS-CoV-2 cases and hospitalizations in the surrounding community.

**Methods:**

Data collected from 9/2021-6/2022 on SARS-CoV-2 cases, hospitalizations, and vaccination rates at the county-week level obtained from the Centers for Disease Control and Prevention were combined with manually-validated, weekly district-level masking policy data obtained from Burbio and district-level school demographic data from the National Center for Education Statistics. Data were analyzed using an event study design, a causal inference method, to estimate the primary outcome: incidence of SARS-CoV-2 cases per 100,000 county residents during the 8-week period following the policy change, stratified by age (in decades). Hospitalization rates per 1,000,000 residents were estimated secondarily.

**Results:**

N= 3,970 districts composed of 53,453 schools and an estimated 31,264,546 students were included. Prior to masking mandate de-adoption, there was no clear trend in case rates in any of the age categories evaluated. Within one-week following policy de-adoption, a small increase in case rates was seen in all age groups evaluated, ranging from 6.92 to 37.34 per 100,000 county residents in the 0-9 year old age group to 25.08 - 120.36 cases per 100,000 residents per week in the 20-49 year old age group. A small increase in hospitalization rates following masking policy de-adoption was seen in all age groups evaluated. Among 0–49-year-olds, increases were seen one week following the policy change. Increases in hospitalization rates were not evident for 50–69-year-olds and >70 year olds until 3 and 4 weeks after the policy change.

**Conclusion:**

Lifting mask mandates in schools were associated with a small increase in case rates in the surrounding community, with the highest increase in individuals of parental age (20–49-year-olds). A small but measurable impact on severe disease, as measured by hospitalization rates, was identified.

**Disclosures:**

**Elissa Schechter-Perkins, MD, MPH**, Gilead Sciences: Grant/Research Support **Westyn Branch-Elliman, MD, MMsc**, DLA Piper, LLC/Medtronic: Advisor/Consultant|DLA Piper, LLC/Medtronic: Expert Testimony|Gilead Sciences: Grant/Research Support

